# Effect of 12‐week continuous positive airway pressure therapy on glucose levels assessed by continuous glucose monitoring in people with type 2 diabetes and obstructive sleep apnoea; a randomized controlled trial

**DOI:** 10.1002/edm2.148

**Published:** 2020-08-08

**Authors:** Anne Margareta Banghøj, Christoffer Krogager, Peter Lommer Kristensen, Klavs Würgler Hansen, Esben Laugesen, Jesper Fleischer, Simon Lebech Cichosz, Per Løgstrup Poulsen, Martin Glymer Kirkegaard, Birger Thorsteinsson, Lise Tarnow

**Affiliations:** ^1^ Department of Endocrinology and Nephrology Nordsjællands Hospital Hillerød Denmark; ^2^ Department of Clinical Medicine Aarhus University Aarhus Denmark; ^3^ Diagnostic Centre Regional Hospital Silkeborg Denmark; ^4^ Department of Endocrinology and Internal Medicine Aarhus University Hospital Aarhus Denmark; ^5^ Steno Diabetes Center Aarhus Aarhus Denmark; ^6^ Steno Diabetes Center Sjælland Holbæk Denmark; ^7^ Department of Health Science and Technology Aalborg University Aalborg Denmark; ^8^ Sleep Disorders Clinic Elective Surgery Centre Regional Hospital Silkeborg Silkeborg Denmark; ^9^ Department of Clinical Medicine Faculty of Health and Medical Sciences University of Copenhagen Copenhagen Denmark; ^10^ Department of Clinical Research Nordsjællands Hospital Hillerød Denmark

**Keywords:** continuous glucose monitoring, continuous positive airway pressure, obstructive sleep apnoea, type 2 diabetes

## Abstract

**Aim:**

Obstructive sleep apnoea (OSA) is frequent in type 2 diabetes (T2D). The aim was to investigate the effect of a 12‐week treatment with continuous positive airway pressure (CPAP) on glycaemic control assessed by continuous glucose monitoring (CGM), HbA1c and fasting blood glucose in patients with T2D and newly detected OSA.

**Methods:**

In a randomized controlled multicentre study, 72 participants with T2D and moderate to severe OSA (78% male, age 62 ± 7, AHI 35 ± 15) were recruited from outpatient clinics in three Danish hospitals and were randomized to CPAP intervention or control. The main outcome was glycaemic control assessed by 6 days CGM at baseline and after 12‐week therapy, as well as by HbA1c and fasting blood glucose.

**Results:**

No significant changes were found in average glucose levels, time in glucose range, time with hypoglycaemia, time with hyperglycaemia or coefficient of variability. HbA1c decreased 0.7 mmol/mol (0.07%; *P* = .8) in the CPAP group and increased 0.8 mmol/mol (0.08%; *P* = .6) in the control group (intergroup difference, *P* = .6). Fasting blood glucose increased by 0.2 mmol/L (*P* = .02) in the CPAP group and by 0.4 mmol/L (*P* = .01) in the control group (intergroup difference, *P* = .7). In a prespecified subgroup analysis comparing participants with high adherence (minimum usage of four hours/night for 70% of all nights) to CPAP to the control group, no significant changes were observed either, although these participants had a tendency towards better glycaemic indices.

**Conclusions:**

CPAP treatment for 12 weeks does not significantly change glycaemic control in patients with type 2 diabetes and OSA.

## Graphical abstract

Former studies show a high prevalence of obstructive sleep apnoea (OSA) in people with type 2 diabetes (T2D). Previous data are divergent on whether continuous positive airway pressure (CPAP) therapy improves glycaemic control in people with T2D and OSA. In this randomized controlled study, no effect was found of 12‐week CPAP treatment on glycaemic control measured by continuous glucose monitoring, HbA1c and fasting blood glucose. This study does not support implementation of routine CPAP to people with T2D and OSA with the purpose of improving glycaemic control.

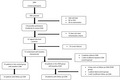

## 1 INTRODUCTION

Obstructive sleep apnoea (OSA) is characterized by recurrent episodes of apnoea (interruption in airflow for at least 10 seconds) and/or hypopnoea (decrease in airflow for at least 10 seconds) during sleep.^1^ OSA is associated with excessive daytime sleepiness, fatigue, problems with concentration, short‐term memory and learning abilities.^2^ The most common treatment for OSA is continuous positive airway pressure (CPAP) therapy. The pressure in the therapy mask increases airflow which leads to a decreased number of airway collapses, thereby improving the effective sleep time and sleep quality.

During recent years, a remarkably high prevalence of OSA is documented in people with type 1^3^ and type 2 diabetes (T2D). The reported OSA prevalence in T2D ranges from 21% to 87%.^4^, ^5^, ^6^ In comparison, estimates of the prevalence of OSA with symptoms in the general population have ranged from 1% to 5% in adult men and 1 to 2% in adult women in older studies.^7^, ^8^

Sleep fragmentation and intermittent hypoxia are hallmarks of OSA. It has previously been shown that insulin sensitivity is compromised when circadian rhythm—for example induced by OSA—is disturbed^9^ and short‐term, intermittent hypoxia is induced^10^ in healthy subjects. Hypoxia results in increased insulin resistance and population studies have shown that OSA is independently associated with insulin resistance and glucose intolerance.^11^ It has been speculated that the insulin resistance in OSA is due, not only to visceral obesity, but also to increased sympathetic drive from the frequent arousals, hypoxia and sleep fragmentation.^12^

A systematic review and meta‐analysis from 2015 by Feng et al^13^ including two randomized controlled trials and four observational studies investigated whether CPAP treatment improves glycaemic control and insulin sensitivity in people with OSA and T2D. The authors concluded that CPAP did not improve the glycaemic control accessed by HbA1c levels but may improve insulin sensitivity. In two of the studies^14^, ^15^ from the systematic review, continuous glucose monitoring (CGM) was used to assess CPAP treatment effect on glycaemic parameters, including glycaemic variability (GV), which adds additional risk beyond HbA1c in the development of micro‐ and macrovascular disease.^16^, ^17^ The advantage of using CGM and not only HbA1c is that changes in glucose levels are revealed faster and more detailed than with HbA1c. This is useful especially at night where the intervention treatment takes place. In all, four studies used CGM; Babu et al^14^ and Guo et al^18^ reported a significantly reduced mean glucose and reduced GV after CPAP use. Dawson et al^15^ also demonstrated a reduced mean glucose but no significant change in variability. A more recent study from 2017 by Morariu et al^19^ showed no change in mean glucose measured by CGM after CPAP use. GV data were not reported.

Thus, existing data are divergent, and the purpose of the present study was therefore to test whether CPAP treatment influences glycaemic control in terms of changes in CGM, HbA1c and fasting blood glucose in people with T2D and newly diagnosed OSA.

## 2 PARTICIPANTS AND METHODS

### 2.1 Design

We performed a randomized controlled multicentre study of intervention with CPAP treatment vs no CPAP. The treatment period included 12 weeks intervention with a subsequent 9‐month open extension.

The primary end‐point of the study was change in carotid‐femoral pulse wave velocity (to be reported elsewhere), and power calculations and sample size calculations were based hereupon. The main end‐point reported in this paper is change in different measures of glycaemic control assessed by CGM and HbA1c.

The regional Committee on Biomedical Research Ethics (no. 1‐10‐72‐232‐14) approved the study, which was registered at the Danish Data Protection Agency. Clinicaltrials.gov identifier NCT02482584.

### 2.2 Participants

Study participants were recruited from the outpatient clinics at three Danish hospitals: Nordsjællands Hospital, Aarhus University Hospital and Regional Hospital Silkeborg. Patients with T2D were contacted by letter between April 2015 and August 2017 and invited to participate in the study. In Denmark, only patients with ‘complicated’ T2D are attending a hospital‐based outpatient clinic. Subjects who agreed to participate were screened with the ApneaLink+^®^ device (ResMed). The definition of complicated T2D can vary, but in Denmark, complicated T2D is loosely defined as T2D with multiple late diabetic complications or/and glycaemic dysregulation (high Hba1c or problems with hypoglycaemia).

Inclusion criteria were age >18 years, diagnosis of T2D (according to WHO criteria) for at least one year, OSA defined by an apnoea‐hypopnoea index (AHI) >15 events/h and signed informed consent. Exclusion criteria were contraindications to CPAP treatment, other sleep breathing disorders (including central sleep apnoea), working in a transportation‐related industry, treated blood pressure >160/95 mm Hg, heart failure (NYHA Class III or IV), atrial fibrillation, C‐peptide <300 pmol/L or HbA1c <53 mmol/mol (7.0%) or >86 mmol/mol (10%). Changes in antidiabetic treatment were not allowed within 4 weeks prior to first visit and during the 12 weeks intervention period.

Patients with sleepiness have a clear indication for CPAP treatment; however, the average waiting time after referral from a general practitioner to a sleep clinic can be up to 3 months in Denmark. Because of this, we considered it acceptable to enrol subjects in a 3 months study period before initiation of CPAP therapy.

### 2.3 The ApneaLink^®^+ screening device

Pulse oximetry, nasal respiratory flow and thorax movements were measured with the ApneaLink^®^+ device (ResMed), that uses the same algorithm as its predecessor ApneaLink^®^.^20^ Participants were instructed by trained study coordinators who provided them with oral and written instructions on how to mount the device at bedtime. After one night's sleep, the equipment was returned to the clinic. Data were downloaded and analysed automatically by computer software. Only data available for more than four hours were accepted for the diagnosis. Apnoea was defined as interruption in airflow for at least 10 seconds and hypopnoea as a decrease in airflow for at least 10 seconds combined with ≥3% desaturation compared to pre‐event baseline during sleep.^1^ The frequency of obstructive events is reported as an index based on the average number of apnoeas and hypopnoeas per hour of sleep, AHI. An AHI score ≥5 events/h is defined as mild OSA, moderate OSA is defined as AHI >15 and ≤30 events/h and severe OSA as AHI >30 events/h.

All screened subjects received a phone call from one of the investigators with information on the results of the screening. Patients with moderate or severe OSA were invited to participate in the main study if all inclusion and no exclusion criteria were met. Possible candidates were provided with information about the study and appropriate time to consider their participation was given. Participants gave a separate written informed consent for the intervention study. All screened subjects with sleep breathing disorders, which were not included in the study, were referred to a sleep clinic.

### 2.4 Continuous glucose monitoring

CGM was performed using a blinded CGM (iPro2 [Medtronic]) with an Enlite sensor (Medtronic) for six consecutive days before randomization and at the end of the 12‐week intervention period. Participants were instructed to complete at least four blood glucose measurements per day with the provided blood glucose meter Contour XT (Bayer). The data from the sensor were downloaded using Medtronics Carelink iPro. The downloaded data contained information on parameters like sensor use, average glucose level (24 hours, daytime and night‐time), time in glucose range defined as 3.9 to 10.0 mmol/L (24 hours, daytime and night‐time), per cent of time in hypoglycaemia defined as a glucose level <3.9 mmol/L, number of hypoglycaemic episodes (defined as glucose <3.9 mmol/L for more than 10 minutes), per cent of time in hyperglycaemia defined as glucose levels above 10.0 and 13.9 mmol/L and coefficient of variability, a measure of oscillations in blood glucose levels (24 hours, daytime and night‐time). CGM data covering at least 75% of the sensor time were considered sufficient.^16^

### 2.5 Clinical investigations

Fasting blood samples were collected from the patients before and after the intervention including HbA1c (analysed by high performance liquid chromatography), blood glucose, plasma total‐, HDL‐ and LDL‐cholesterol, and creatinine. Overnight urine samples were collected to measure albumin and creatinine.

Clinical measures were collected at baseline and after 12 weeks including hip, waist and neck circumferences (measured at the widest point), body weight and height and blood pressure measured twice by an oscillometric method (Microlife WatchBP Office, Microlife AG).

### 2.6 Diabetic complications

Nephropathy was defined as microalbuminuria (urinary albumin/creatinine ratio 30‐299 mg/g) or macroalbuminuria (urinary albumin/creatinine ratio ≥300 mg/g).

Peripheral neuropathy was diagnosed based on monofilament testing, classifying peripheral neuropathy as lack of sensation in at least two out of three touches. Test of vibration sensation was not done on all sites. Therefore, the monofilament data were reported.

Signs of autonomic neuropathy were investigated in the fasting state by the Vagus™ device (Medicus Engineering Aps)^21^ which obtains an electrocardiogram recording by two handhold electrodes during rest and three active tests. One abnormal test is indicative of early signs of cardiac autonomic neuropathy (CAN), referred to as borderline CAN. Two or more abnormal tests indicate established CAN. All heart rate‐based tests were evaluated according to age‐dependent cut‐off levels.^22^

### 2.7 Questionnaires

Epworth Sleepiness Scale (ESS) measures daytime sleepiness^23^ on a scale from 0 to 24. The total score is calculated from 8 questions rating the chances of dozing off or falling asleep in eight different situations commonly encountered in daily life. A score above 10 is abnormal and indicates high level of sleepiness.^23^ People were randomized regardless if ESS were high or low. Patients filled in the ESS questionnaire at baseline and after 12 weeks. The ESS is commonly used for quantifying symptoms of sleepiness, and ESS score increases with severity of OSA, at least in the general population.^24^

### 2.8 Intervention

Randomization was performed using a computer‐generated balanced block, with randomly varying block sizes of four, six or eight subjects, and participants were randomized 1:1 to either CPAP treatment or control.

Participants randomized to the CPAP group received an Airsense 10 (ResMed) CPAP device. Subjects from Aarhus University Hospital and Regional Hospital Silkeborg were referred to the outpatient Clinic for Sleep Disorders at Regional Hospital Silkeborg for instructions on how to use the device, whereas subjects from Nordsjællands Hospital were instructed in the diabetes outpatient clinic. All patients were instructed by a trained nurse initially, after one month and after 12 weeks of initiation of the CPAP therapy. Different mask types—full face or nasal mask—in different sizes were fitted to the participants until treatment was well‐tolerated. Good compliance to the CPAP device was defined as a minimum usage of four hours per night for at least 70% of all nights.^25^

Participants randomized to the control group were invited to follow‐up visits after 1 month and after 12 weeks or order to follow the same protocol as the intervention group. The control group received no treatment other than recommendations on sleep hygiene. All participants in the control group refrained from seeking treatment for their OSA in the intervention period.

### 2.9 Statistical analysis

Data are presented as mean ± SD for normally distributed variables or as median and interquartile range for skewed data. Comparisons between groups at baseline—including CGM variables—were performed with Student's *t* test, paired *t* test and Mann Whitney *U* test as appropriate. Categorical parameters were compared with a chi‐square test. The effect of 12‐week CPAP treatment on glycaemic parameters was analysed by comparing the delta values (visit 12 weeks minus baseline) in the two groups of different CGM variables using Student's *t* test. Calculations were done on all glucose values (24 hours) and separately for night (24:00 to 06:00) and day (6:00 to 24:00). To further assess the effect of CPAP vs control on our end‐points, we performed a mixed general linear model (analysis of covariance [ANCOVA]; SPSS: Analyze → General Linear Model → Univariate), which make it possible to adjust the results for each continuous end‐point for confounding. Treatment (CPAP vs control) was regarded as a fixed factor and baseline Hba1c, AHI‐score, and time in range (and other related glycaemic variables, ie CV of glucose) was regarded as covariates, since these variables could potentially bias an effect of the CPAP treatment on the different glycaemic variables. A two‐tailed *P*‐value of ≤.05 was considered statistically significant. All statistical analyses were performed using SPSS 25.0 (SPSS).

## 3 RESULTS

### 3.1 Patient characteristics

**Figure 1:**
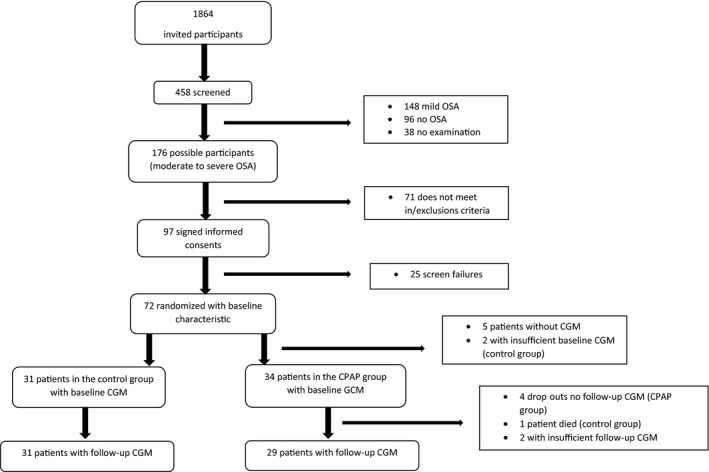
Patient flow chart. The screening procedure was slightly different at the sites, since the Aarhus and Silkeborg investigators assessed the possible participants according to the in‐/exclusion criteria before sending out letters

A total of 458 of 1864 invited patients (25%) participated in the screening for OSA (Figure 1). Among these, 38 patients (8%) were unable to complete the overnight registration due to misunderstanding of instructions or discomfort with the device and were therefore not included. Hence, 420 participants completed the screening. Hereof, 96 (23%) had no OSA, 148 (35%) had mild OSA (AHI ≥5 and <15 events/h) and 176 (42%) had moderate OSA (AHI ≥ 15 and ≤30 events/h) or severe OSA (AHI >30 events/h).

Of the 176 eligible candidates, 97 accepted to participate in the study. After signing the informed consent form, 25 participants were excluded due to low C‐peptide or low HbA1c, heart failure, atrial fibrillation or uncontrolled hypertension. A total of 72 participants were randomized, 65 of them completed the 12‐week intervention, six patients discontinued the study primarily due to incompatibility to the CPAP device and one patient in the control group died unexpectedly (Figure 1). The baseline characteristics of the 72 participants are presented in Table 1. Participants were predominantly male, and at baseline characteristics were comparable between the intervention and the control group, except for BMI which was significantly higher in the intervention group. There were no differences in baseline characteristics between the participants who discontinued the study and participants who completed the study (data not shown).

**Table 1:** Baseline characteristics

	CPAP n = 36	Control n = 36	*P*‐value
Male sex %	72%	83%	.26
Age (y)	63 ± 7	61 ± 8	.24
Duration of diabetes (y)	16 ± 7	16 ± 10	.97
Height (m)	1.74 ± 0.09	1.73 ± 0.07	.72
Body weight (kg)	109 ± 16	99 ± 11	.004
Body mass index, BMI (kg/m^2^)	36.1 ± 4.7	33.1 ± 3.5	.003
Waist circumference (cm)	119 ± 17	115 ± 8	.21
Hip circumference (cm)	118 ± 12	112 ± 11	.02
Neck circumference (cm)	47 ± 14	44 ± 3	.25
Systolic blood pressure (mm Hg)	141 ± 15	141 ± 16	.98
Diastolic blood pressure (mm Hg)	81 ± 9	82 ± 8	.47
Sleep related data			
Epworth Sleepiness Scale (ESS)	7 ± 3	8 ± 5	.24
Apnoea‐hypopnoea index (AHI)	32 (IQR 21‐43)	34(IQR 22‐49)	.62
Laboratory values			
HbA1c (mmol/mol)	64 ± 9 (8.0 ± 0.8)	65 ± 9 (8.1 ± 0.8)	.81
Plasma fasting glucose (mmol/L)	10.0 ± 2.6	9.1 ± 2.1	.11
Plasma creatinine (µmol/L)	85.5 ± 35.0	83.6 ± 20.2	.77
Plasma total‐cholesterol (mmol/L)	4.2 ± 0.9	3.9 ± 0.9	.26
Plasma LDL‐cholesterol (mmol/L)	2.1 ± 0.8	1.8 ± 0.7	.16
Plasma HDL‐cholesterol (mmol/L)	1.17 ± 0.24	1.16 ± 0.35	.89
Lifestyle			
Alcohol consumption per week (0‐7 units, 8‐21 units, >21 units)	84%/8%/8%	89%/11%/0%	.20
Current smokers, n (%)	3 (8%)	7 (19%)	.38
Late diabetic complications			
Nephropathy			
Normoalbuminuria	63%	64%	.60
Microalbuminuria	34%	27%	
Macroalbuminuria	3%	9%	
Autonomic neuropathy			
Normal	24%	38%	
Borderline CAN^a^	33%	24%	
Established CAN^a^	43%	38%	.45
Peripheral neuropathy, n (%)	5 (14%)	3 (8%)	.43
Cardio vascular disease (n)	AMI = 2	AMI = 3	–
	TCI = 6	TCI = 4	–
Use of medication			
Metformin	83%	78%	.55
Other oral antidiabetic drugs (DPP‐4/SGLT‐2/SU)	23%	31%	.69
Insulin	61%	69%	.46
GLP‐1 agonist	58%	50%	.48
Antihypertensive treatment	94%	83%	.13
Diuretics	58%	47%	.35
Statin treatment	79%	83%	.59
Aspirin	53%	51%	.91

^a^ Cardiovascular autonomic neuropathy.

The participants had moderate to severe OSA, median AHI 32 (IQR 22‐45) events/h. They were largely asymptomatic and did not report sleepiness, ESS 7 ± 4 (range 0‐23) points, 16% of the patients had an ESS above 10 points.

### 3.2 CPAP treatment

The average use of the CPAP device was 5.6 ± 1.9 hours per night. Good compliance was obtained by 16 participants (44%), that is they used CPAP for more than 4 hours more than 70% of the nights. In the last month of the CPAP intervention, the average use was 5.4 ± 2.1 hours per night and during this period 15 participants (42%) had good compliance. The average use for the compliant participant was 6.9 (range 5.3 to 8.4) hours per night. For the participants noncompliant to CPAP, the average use was 3.9 (range 1.2 to 6.2) hours per night.

Participants with good adherence to CPAP did not differ from the control group at baseline in respect to diabetes duration, blood pressure, HbA1c, laboratory values, ESS and AHI, except for BMI which was still significantly higher in the compliant CPAP group.

### 3.3 AHI and ESS

The AHI was reduced from 32 (IQR 22‐45) events/h to 0.7 (IQR 0.4‐1.1) events/h for subjects randomized to treatment. ESS was 7 ± 3 points in the CPAP group at baseline and 5 ± 4 points after 12 weeks (*P* = .003, paired t test). In the control groups, ESS was 8 ± 5 points at baseline and 7 ± 5 points after 12 weeks (*P* = .03, paired t test). Delta values of ESS in the two groups were not different (*P*‐value .8).

### 3.4 Continuous glucose monitoring

In average, patients used the CGM for 151 hours at baseline and at the 12 weeks follow‐up both in the CPAP group and control group. All patients had sufficient CGM data except for two participants at baseline and another two participants after the 12 weeks’ intervention, hence the different number of participants in Tables 2,3 and 4. No difference between the two groups was observed at baseline for any of the CGM derived data. Similarly, no differences between groups after intervention in mean glucose (24 hours, day and night), different measures of being in/out of range or GV were observed. The ANCOVA analysis adjusted for baseline BMI, HbA1c, AHI and baseline value of the dependent variable did not show statistically significant effects on glycaemia during CPAP treatment (Tables 2,3 and 4).

**Table 2:** Continuous glucose monitoring data (CGM) in 65 patients with type 2 diabetes and obstructive sleep apnoea (OSA) randomized to 12 weeks treatment with continuous positive airway pressure (CPAP) or no CPAP

All patients	CPAP/baseline n = 34	CPAP/12 wk n = 29	Control/baseline n = 31^a^	Control/12 wk n = 31^a^	CPAP change n = 29	*P*‐value paired *t* test	Control group change n = 29^a^	*P*‐value paired *t* test	Between‐group difference^b^	*P*‐value
Time with CGM‐data (%)	97.9 ± 5.4	97.9 ± 4.6	98.8 ± 2.2	97.1 ± 5.5	0.1 ± 7.8	1.0	−1.1 ± 5.1	.2	1.2	.5
Average glucose, 24 h (mmol/L)	9.8 ± 1.8	9.5 ± 1.5	9.4 ± 1.9	9.8 ± 2.0	−0.2 ± 1.8	.6	0.5 ± 2.3	.2	−0.7	.2
Average glucose, night (mmol/L)	9.1 ± 2.0	8.8 ± 1.7	8.4 ± 2.0	9.1 ± 1.9	−0.1 ± 1.9	.7	0.6 ± 2.3	.2	−0.7	.2
Average glucose, day (mmol/L)	10.0 ± 1.8	9.7 ± 1.5	9.7 ± 1.9	10.1 ± 2.1	−0.2 ± 1.9	.6	0.5 ± 2.4	.3	−0.7	.2
Time in range (3.9‐10.0 mmol/L; %)										
24 h	59.7 ± 22.4	63.1 ± 20.3	64.4 ± 23.6	57.7 ± 25.0	0.04 ± 20.6	.9	−6.6 ± 27.3	.2	6.6	.3
Night	65.3 ± 24.7	67.7 ± 24.0	71.1 ± 24.2	62.7 ± 26.4	0.5 ± 20.8	.9	−7.4 ± 28.6	.2	7.9	.3
Day	56.7 ± 23.0	60.7 ± 20.5	61.0 ± 25.0	55.1 ± 26.1	0.2 ± 24.1	.9	−6.2 ± 29.3	.3	6.4	.4
Glucose < 3.9 mmol/L (% of time)	0.9 ± 1.7	0.6 ± 1.5	0.8 ± 1.6	0.9 ± 2.4	−0.5 ± 2.3	.3	−0.1 ± 2.8	.9	−0.4	.6
Hypoglycaemic episodes (no.)^c^	0.6 ± 1.1	0.6 ± 1.8	0.8 ± 1.5	0.7 ± 1.8	−0.1 ± 1.8	.8	−0.3 ± 2.2	.6	0.2	.7
Glucose > 10.0 mmol/L (% of time)	39.4 ± 23.0	36.3 ± 20.7	34.9 ± 24.0	41.4 ± 25.6	−0.5 ± 21.3	.9	6.7 ± 27.9	.2	−7.2	.3
Glucose > 13.9 mmol/L (% of time)	10.2 ± 13.9	7.5 ± 8.0	7.7 ± 11.7	10.5 ± 16.5	−2.4 ± 12.6	.3	3.0 ± 18.8	.4	−5.4	.2
Glycaemic variability										
CV, 24 h	0.25 ± 0.06	0.25 ± 0.05	0.24 ± 0.07	0.24 ± 0.07	−0.01 ± 0.05	.2	−0.004 ± 0.08	.7	−0.006	.7
CV, night	0.22 ± 0.08	0.23 ± 0.08	0.21 ± 0.08	0.21 ± 0.09	0.006 ± 0.09	.7	0.006 ± 0.11	.8	−0.01	.7
CV, day	0.25 ± 0.06	0.25 ± 0.05	0.23 ± 0.07	0.23 ± 0.07	−0.01 ± 0.05	.1	−0.003 ± 0.08	.9	−0.01	.5

Abbreviation: CV, coefficient of variation.

^a^ Two patients from baseline and two patients at 12 wk follow‐up had missing CGM data (not the same patients), as a result only 29 patients in group change.

^b^ In the between group difference column, the control group is reference.

^c^ Episode of hypoglycaemia is defined as glucose <3.9 mmol/L for more than 10 min.

**Table 3:** Continuous glucose monitoring data (CGM) only in the compliant CPAP users, defined as a minimum usage of 4 h per night for at least 70% of all nights

Only compliant CPAP users	CPAP/baseline n = 16	CPAP/12 wk n = 16	CPAP group change n = 16	*P*‐value paired *t* test	Between group difference^a^ CPAP n = 16 control = 29	*P*‐value
Time with CGM‐ data (%)	97.1 ± 7.5	98.8 ± 2.2	1.7 ± 7.9	.4	2.8	.2
Average glucose, 24 h (mmol/L)	9.9 ± 2.0	9.5 ± 1.1	−0.4 ± 1.7	.3	−0.9	.2
Average glucose, night (mmol/L)	9.5 ± 2.3	8.9 ± 1.2	−0.7 ± 1.9	.2	−1.3	.07
Average glucose, day (mmol/L)	10.1 ± 2.0	9.7 ± 1.1	−0.4 ± 1.7	.4	−0.9	.2
Time in range (3.9‐10.0 mmol/L; %)						
24 h	59.2 ± 22.3	64.3 ± 17.4	5.1 ± 16.0	.2	11.7	.1
Night	62.2 ± 28.8	69.5 ± 21.3	7.3 ± 21.3	.2	14.7	.08
Day	57.4 ± 20.5	61.4 ± 17.7	4.0 ± 17.3	.4	10.2	.2
Glucose <3.9 mmol/L (% of time)	0.9 ± 1.8	0.2 ± 0.6	−0.6 ± 1.7	.1	−0.5	.5
Hypoglycaemic episodes (no.)^b^	0.6 ± 1.2	0.3 ± 0.7	−0.3 ± 0.9	.2	−0.04	1.0
Glucose >10.0 mmol/L (% of time)	40.0 ± 22.8	35.5 ± 17.4	−4.5 ± 16.5	.3	−11.2	.2
Glucose >13.9 mmol/L (% of time)	12.6 ± 17.6	7.0 ± 7.7	−5.6 ± 15.5	.2	−8.6	.1
Glycaemic variability						
CV, 24 h	0.27 ± 0.06	0.25 ± 0.06	−0.02 ± 0.04	.1	−0.02	.6
CV, night	0.24 ± 0.08	0.22 ± 0.09	−0.01	.5	−0.16	.5
CV, day	0.26 ± 0.06	0.25 ± 0.06	−0.02	.1	−0.01	.5

Note

CV, coefficient of variation.

^a^ In the between group difference column, the control group is reference.

^b^ Episode of hypoglycaemia is defined as glucose <3.9 mmol/L for more than 10 min.

**Table 4:** Mixed general linear model of the association between different CGM variables (the dependent variables) and CPAP treatment vs control (univariate analysis) with baseline values of Hba1c, AHI, BMI and dependent variable as covariates (multiple analysis) in 58 patients with type 2 diabetes and obstructive sleep apnoea randomized to CPAP or not CPAP for 12 wks

Variable	Regression coefficient (95% CI)	*P*‐value	Regression coefficient (95% CI)	*P*‐value
	Effect of CPAP vs control control = reference		Effect of CPAP vs control adjusted for baseline Hba1c, AHI, BMI and dependent variable Control = reference	
All 58 participants				
Average CGM glucose, 24 h (mmol/L)	−0.7 (−1.8 to 0.4)	.22	−0.8 (−1.7 to 0.2)	.10
Average CGM glucose night (mmol/L)	−0.7 (−1.8 to 0.4)	.21	−0.5(−1.5 to 0.4)	.27
Average CGM glucose day (mM)	−0.7 (−1.8 to 0.5)	.25	−0.9 (−1.9 to 0.1)	.08
Time in range (3.9‐10.0 mmol/L; %)				
24 h	+6.6 (−6.2 to 19.3)	.31	+ 9.2 (−2.6 to 21.0)	.12
Night	+6.9 (−6.3 to 20.1)	.30	+ 5.5 (−7.0 to 18.0)	.38
Day	+6.3 (−7.8 to 20.4)	.37	+ 10.8 (−2.0 to 23.6)	.10
Glycaemic variability, coefficient of variation	−0.008 (−0.05 to 0.03)	.69	−0.005 (−0.03 to 0.04)	.77
dCPAP noncompliant participants excluded				
Average CGM glucose, 24 h (mmol/L)	−1.0 (−2.3 to 0.4)	.15	−0.5 (−1.7 to 0.7)	.40
Average CGM glucose night (mmol/L)	−1.2 (−2.6 to 0.1)	.07	−0.1 (−1.2 to 1.0)	.86
Average CGM glucose day (mmol/L)	−0.9 (−2.2 to 0.5)	.21	−0.6 (−1.9 to 0.6)	.30
Time in range (3.9‐10.0 mmol/L; %)				
24 h	+11.7 (−3.4 to 26.7)	.13	+ 8.8 (−5.7 to 23.3)	.23
Night	+14.7 (−1.8 to 31.2)	.08	+ 3.3 (−11.8 to 18.4)	.66
Day	+10.2 (−6.0 to 26.4)	.21	+ 11.1 (−4.4 to 26.6)	.16
Glycaemic variability, coefficient of variation	−0.011 (−0.06 to 0.04)	.64	−0.008 (−0.06 to 0.04)	.73

Note

In the lower part of the table, CPAP noncompliant participants are excluded leaving 16 compliant participants and 31 control participants for analysis.

Abbreviations: CGM, continuous glucose monitoring; CPAP, continuous positive airway pressure.

The prespecified subgroup analysis on the fully compliant participants did not differ from the main findings, although these 16 participants had a tendency towards better glycaemic indices during night. Change in average glucose night‐time: CPAP group −0.7 ± 1.9 mmol/L vs control group + 0.6 ± 2.3 mmol/L, *P* = .07. Change in time in range night‐time; CPAP group + 7.3 ± 21% vs −7.4 ± 29%, *P* = .08.

### 3.5 Glycaemic control

HbA1c did not change significantly in the two groups during the 12 study weeks. In the CPAP group (n = 31), HbA1c was 64 ± 9 mmol/mol (8.0 ± 0.8%) at baseline and 63 ± 10 mmol/mol (7.9 ± 0.9%) at 12 weeks (*P* = .8, paired t test). In the control group (n = 31), HbA1c was 65 ± 8 mmol/mol (8.1 ± 0.8%) at baseline and 66 ± 12 mmol/mol (8.2 ± 1.1%) at 12 weeks (*P* = .6, paired *t* test). Delta values of HbA1c in the two groups were not significantly different. When analysing only compliant CPAP users, HbA1c in the CPAP group decreased by 0.4 mmol/mol (0.04%; *P* = .9, paired t test). Fasting blood glucose was 9.7 ± 2.8 mmol/L in the CPAP group (n = 30) at baseline and 9.9 ± 2.5 mmol/L after 12 weeks (*P* = .02, paired *t* test). In the control group (n = 30), fasting blood glucose was 9.1 ± 2.1 mmol/L at baseline and 9.5 ± 2.4 mmol/L after 12 weeks (*P* = .01, paired *t* test). Delta values of fasting blood glucose in the two groups were not significantly different.

Adjustments of the participants’ blood glucose lowering medication was not allowed during the 12 weeks trial, nevertheless four patients in the CPAP group and two in the control group experienced intensification of their treatment prescribed by their general practitioner.

### 3.6 Blood pressure and BMI

There was no significant change in systolic or diastolic blood pressure during the 12 weeks study, data not shown. Furthermore, there was a significant decrease in BMI in the control group (−0.3 ± 0.2, *P* = .05) and no change was found in the intervention group.^26^

## 4 DISCUSSION

In this randomized controlled study, no effect was found of 12 weeks CPAP treatment on CGM‐based average glucose levels, time in glucose range, time with hypoglycaemia, time with hyperglycaemia or GV measures in people with T2D. This was true both during night and day and when adjusting for HbA1c, BMI and AHI. Neither did we find any effect among compliant CPAP users although these participants had a tendency towards better glycaemic indices. The results from the subgroup analyses are with reservations since a limited number of participants are included in the analyses.

There are four previous prospective trials investigating the effect of CPAP treatment on glycaemic control evaluated by CGM in people with T2D.^14^, ^15^, ^18^, ^19^ In general, these studies have limitations like absence of a control group, a limited number of participants and short duration of CPAP treatment. Thus, Babu et al,^14^ Dawson et al^15^ and Guo et al^18^ studied the effect of CPAP treatment for 83, 41 and 30 days, respectively, in 25, 20 and 40 patients and all three studies were without a control group. They all demonstrated improvements in CGM indices after CPAP treatment. A more recent study by Morariu et al^19^ lasting 30 days and including a small number of patients (CPAP group n = 12, sham CPAP group n = 11) found no improvement in CGM indices.

Our study had a 12‐week intervention period, but the adherence to the treatment was challenging and only 16 of the 36 participants (44%) randomized to the intervention had good compliance. Our participants did not feel sleepy at baseline (ESS score was below 10) which could be one explanation for the low compliance. Former studies^27^, ^28^ shows conflicting results when comparing adherent CPAP users with nonadherent in terms of ESS values. Kohler et al^27^ found that ESS was not associated with long‐term compliance whereas Jacobsen et al^28^ found that ESS was independently associated with CPAP adherence. Lack of compliance to CPAP treatment is not a problem unique to our study. Westlake et al^29^ presented a study comparing adherence to CPAP in people with T2D and patients attending a sleep clinic without T2D. In both groups, compliance after 3 months and 1 year was around 40% and thus comparable to the present study. Therefore, low adherence is a general limitation of CPAP therapy. In a study by Mokhlesi et al^30^ found that one week of CPAP use, in average 7.9 hours, improved 24‐hour profile of glucose; however, this study was performed in laboratory which makes it difficult comparing it with studies that have longer time of duration and where CPAP treatment takes place at home. Babu et al,^14^ Dawson et al^15^ and Guo et al^18^ all report daily CPAP use from 4.2 ± 2.9 hours per night to 5.8 ± 1.0 hours per night which is comparable with the results from this study.

A review from 2016 by Martínez‐Cerón et al^31^ and another review by Labarca et al^32^ from 2018 investigated the effect of CPAP treatment on glucose metabolism in patients with OSA, both participants with diabetes and without. Glucose metabolism was evaluated by HbA1c levels, calculation of homeostatic model assessment of insulin resistance (HOMA‐IR) index or by hyperinsulinaemic euglycaemic clamps. The conclusion was indecisive in the Martínez‐Cerón et al^31^ study and in the Labarca et al^32^ study showed that CPAP does not improve glycaemic control in respect to HbA1c and fasting glucose.

The lack of effect of CPAP on glycaemic indices in the present study is surprising since CPAP therapy of OSA is associated with a reduction in sympathetic nervous system (SNS) overactivity.^33^ Activation of the SNS should theoretically lead to a higher blood glucose concentration via stress hormones (eg cortisol and adrenaline) secreted during apnoea periods. Therefore, when the activity of the SNS is reduced by CPAP therapy, we would expect to see a decrease in blood glucose. One explanation could be that the included participants had moderate to severe OSA with an AHI around 30, which contribute to only a minor activation of SNS. In addition, three of four patients in the CPAP group had signs of autonomic neuropathy and therefore likely a disrupted sympathetic response. Unfortunately, we did not directly measure sympathetic activity in this study. Furthermore, since only a few participants in the CPAP group were sufficient adherent to the treatment, the power of our study is reduced, which makes it difficult to detect small differences between the control group and the CPAP group. The tendency of effect of CPAP on the CGM‐derived glycaemic variables in the compliant group supports this. Moreover, it cannot be excluded, that participating as a control subject in the study has changed the behaviour of the control participants in relation to food intake, sleep patterns and physical exercise. Such changes could potentially diminish the difference between the groups. However, if any changes in lifestyle or behaviour took place in the control group, these changes did not translate into changes in body weight, HbA1c or glycaemic indices.

Our study has several strengths. It is the largest prospective randomized controlled trial with a study period of 12 weeks, being longer than in previously studies to date. The high‐quality CGM data^16^ which allowed us to have detailed information about both daytime and night‐time fluctuations instead of only HbA1c and fasting blood glucose is also a strength.

Our study has limitations. Despite a rigorously randomization of participants, the two groups differed in BMI, which is unfortunate, since high BMI is a risk factor for developing OSA.^8^ However, the two groups were similar in OSA severity and adjusting for BMI did not change our results. Low adherence to CPAP is common which results in the relatively small number of compliant users. With a larger sample size, the tendency towards better glycaemic control overall and especially during night‐time might have been more distinct. Moreover, the study was not blinded (ie the control group was not a placebo group treated with sham CPAP) since sham CPAP treatment is difficult to apply without unblinding the participants. The inclusion of participants with only moderate to severe OSA and T2D makes it difficult to generalize to other groups. Our study population are predominantly male which is a limitation; however, this also reflects that OSA is largely a disorder that affects males.

One of the goals of treatment of T2D and related disorders, for example OSA, is to reduce morbidity and mortality and to avoid late diabetic complications. One way to avoid complications is good glycaemic control, which we aimed to improve using CPAP treatment. Although CPAP treatment is an accepted treatment modality in OSA due to its effect on symptom scores, quality of life and reduction in AHI,^31^ our study does not support implementation of routine CPAP treatment to people with T2D and OSA with the purpose of improving glycaemic control.

## CONFLICT OF INTEREST

Fleischer J. is the co‐inventor of Vagus™ device and holds stock in Medicus Engineering. No other author has reported conflicts of interest. ResMed Maribo have not had any influence on the design or conducting of the study, nor have they influenced the analysis of data or the manuscript.

## AUTHOR CONTRIBUTION

AMB performed measurements, collected data, performed statistical analysis and wrote the manuscript. CK performed measurements, collected data and reviewed/edited the manuscript. PLK contributed to the discussion, the statistical analysis and reviewed/edited the manuscript. KWH, EL, JF, SLC, PLP and MGK contributed to the discussion and reviewed/edited the manuscript. BT and LT concepted the study, contributed to the discussion and reviewed/edited the manuscript and are guarantors of the study.
